# South African mental health workers’ knowledge and attitudes to trans and gender-diverse people

**DOI:** 10.4102/sajpsychiatry.v32i0.2513

**Published:** 2026-02-04

**Authors:** Maya Jaffer, Laila Paruk, Belinda Marais

**Affiliations:** 1 Department of Psychiatry, Faculty of Health Sciences, University of the Witwatersrand, Johannesburg, South Africa

**Keywords:** transgender healthcare, mental health worker attitudes, psychiatric setting, the Transgender Knowledge, Attitudes, and Beliefs (T-KAB) scale, South Africa

## Abstract

**Background:**

Transgender and gender-diverse (TGD) individuals experience higher rates of mental illness compared to cisgender populations. Accessing appropriate care remains challenging because of discrimination and a lack of provider knowledge. There is limited evidence exploring mental health worker views in a local setting.

**Aim:**

This study aimed to explore knowledge and attitudes of South African mental health workers in specialised psychiatric settings towards TGD people.

**Setting:**

This study was conducted at two specialist tertiary psychiatric hospitals in Johannesburg.

**Methods:**

A cross-sectional descriptive study utilising the Transgender Knowledge, Attitudes, and Beliefs (T-KAB) scale was conducted among 150 mental health workers. Information on socio-demographic features, professional characteristics, and work experience was collected and analysed in relation to T-KAB scores.

**Results:**

Participants demonstrated moderately favourable attitudes with a mean T-KAB score of 2.81/4.00. Professional category significantly influenced attitudes, with psychologists followed by medical doctors scoring the highest, and nurses and social workers scoring lowest. Age, sexual orientation, and religious affiliation were significantly associated with T-KAB scores, while years of practice, previous training in TGD-related care, and prior exposure to TGD patients showed no associations.

**Conclusion:**

Mental health professionals in South African psychiatric hospitals hold moderately positive attitudes towards TGD individuals, with significant variations across professional categories. Prior training and clinical exposure were not associated with attitudes.

**Contribution:**

This study provides one of the first assessments of mental health worker attitudes towards TGD individuals in an African setting. Nurses constitute the majority of the workforce and demonstrate less favourable attitudes, which has implications for approaches to improve transgender-affirming mental healthcare in this setting.

## Introduction

Globally, sexual minority populations, including transgender and gender-diverse (TGD) individuals, experience substantially higher rates of mental health issues compared to cisgender populations, such as depression, anxiety, suicidality and substance use disorders.^1,2,3^ This disproportionate mental health burden stems at least in part from experiences of discrimination, violence, and societal marginalisation, and is commonly attributed to the minority stress model, in which external stressors are transformed into internalised stressors, such as shame, which elevate mental health risks.^3,4^ Meta-analyses confirm that these challenges are substantial, with several studies revealing particularly high rates of major depressive disorders affecting up to one-third of TGD individuals.^1,2,3^ Similar problems are observed in South Africa, with studies reporting markedly high prevalence of mental health problems and psychological distress among TGD individuals.^5,6^ In a large community-led study in 2019, more than half of the participants classified as currently depressed, one-fifth reported severe anxiety, and two-thirds reported lifetime suicidality.^5^

Despite these high burdens, accessing mental health care is fraught with barriers. Many TGD individuals report concerns about being pathologised for their gender identity, previous experiences of discrimination in a health care setting, and other systemic issues of access such as affordability of services.^7,8,9^ A US-based study found that recognisably transgender individuals were twice as likely to report experiences of discrimination in mental healthcare settings.^9^ Local evidence in a South African setting confirms that discrimination in healthcare settings remains a pervasive issue, with patients frequently encountering stigma and moral judgement from providers and sometimes refusal of care.^5,10^ Poor understanding of TGD-specific issues may compromise clinical care, particularly as conditions such as post-traumatic stress disorders, personality disorders and eating disorders, have been found to be more prevalent than in cisgender individuals and may present uniquely in TGD populations.^11,12^ Inpatient psychiatric settings present a particularly important and understudied context for examining attitudes towards TGD individuals, as recent evidence suggests stigma manifests at multiple levels in these environments, from institutional policies and infrastructure to interpersonal interactions between staff and patients.^13^ Given this pervasive evidence on discrimination and barriers to care, understanding provider knowledge and attitudes towards TGD individuals is essential for improving mental healthcare access and quality.

While identifying negative attitudes is important, understanding positive provider attitudes is equally crucial, as they play a pivotal role in improving TGD mental health outcomes. Access to a transgender-inclusive provider has been shown to protect against depression and suicidality.^14^ Recent systematic reviews and studies robustly suggest that mental health workers generally endorse favourable attitudes towards TGD individuals,^15,16,17,18,19^ with socio-demographic factors such as younger age, being in a racial minority, female gender, non-religiosity, and personal acquaintance with lesbian, gay, bisexual, transgender, and queer or questioning (LGBTQ) or transgender individuals being linked to more positive attitudes.^15,16,17,18,20,21^ Implicit biases, which are harder to measure, may still negatively affect the care experience.^17^ However, most of this evidence comes from more developed settings, with limited research examining mental health worker attitudes in African psychiatric contexts.

Alongside socio-demographic determinants of attitudes, attention has been paid to potentially modifiable factors such as training in TGD healthcare and clinical exposure to TGD patients. Global evidence suggests that only a minority of mental health workers receive formal training in gender-affirming care during their studies, with most relying on self-directed learning and professional development opportunities accessed independently after qualifying.^18,19,22^ Evidence suggests that both clinical experience with and exposure to TGD patients can motivate mental health workers to seek further education and can result in more positive attitudes, although findings remain mixed.^15,16^ In addition, systematic reviews have suggested that greater curricular training time on LGBTQ issues has positive effects on knowledge and attitudes.^15^

Turning to the South African setting, a lack of training and preparedness in dealing with TGD issues is frequently cited by healthcare workers, with confidence in caring for patients from sexual minorities remaining low.^10,23^ Although South Africa has published progressive and comprehensive guidelines for gender-affirming care that affirm the important role of mental health providers in supporting TGD individuals,^24^ evidence on mental healthcare provision for this population in a local context remains scarce. Recent systematic reviews of educational interventions in transgender healthcare show promising but mixed results: while training initiatives generally demonstrate improvements in knowledge, attitudes and self-reported competence among healthcare workers, there is no consensus on best practices for education delivery, and limited evidence on long-term impacts.^25^

Addressing these challenges requires an understanding of local healthcare worker attitudes towards TGD patients. While international studies have examined mental health worker attitudes, there is a paucity of evidence from African psychiatric settings, where cultural contexts and training gaps may differ substantially. Identifying personal and professional factors associated with negative attitudes can inform targeted training initiatives and improve service delivery in local psychiatric settings. This study therefore aimed to address this gap in the evidence.

### Aim and objectives

This study aimed to explore the knowledge and attitudes of South African mental health workers practising in a specialised psychiatric hospital setting towards TGD people.

The study objectives were:

- Describe the knowledge and attitudes of mental health workers working in specialist psychiatric hospitals in Johannesburg, South Africa towards TGD individuals.
- Compare differences in knowledge and attitudes towards TGD individuals based on mental health worker demographic characteristics.
- Compare differences in knowledge and attitudes towards TGD individuals based on mental health worker training and experience.

## Research methods and design

### Study design and setting

This cross-sectional descriptive study was conducted at two specialised tertiary psychiatric hospitals in Johannesburg, South Africa: Tara Hospital and Sterkfontein Hospital. Both hospitals are affiliated with the University of the Witwatersrand (Wits) academic circuit. Tara Hospital’s services include general adult psychiatry, neuropsychiatry, child and adolescent psychiatry, an eating disorder unit, and a psychotherapy unit, for both inpatients and outpatients. Sterkfontein Hospital provides general adult psychiatry inpatient services and has a forensic psychiatric unit and a dual diagnosis unit for patients with both substance use disorders and psychiatric disorders.

### Sampling

The study population comprised mental health workers employed at Tara and Sterkfontein hospitals, including professional nurses, medical officers, psychiatry registrars, consultant psychiatrists, clinical psychologists, intern psychologists, occupational therapists (OT), and social workers. At the time of data collection, these hospitals together employed approximately 650 mental health workers across these disciplines. All students, including medical, psychology, and nursing, were excluded. A convenience sampling approach was used to recruit participants from the eligible clinical staff population.

### Data collection and study measures

Data collection took place between June 2024 and November 2024. The survey was made available in both paper and electronic formats. Paper surveys were distributed at both hospitals with designated collection boxes for completed surveys. An electronic version of the survey, hosted on the REDCap data management platform, was distributed via email to clinical staff and could also be accessed through quick-response (QR) code links displayed on posters throughout both hospitals. Both formats were offered to maximise participation, anticipating potential participants may have different preferences for survey completion, but the content and presentation of questions were identical across formats. Participation was voluntary for all staff members.

The survey consisted of three sections: The first section collected socio-demographic information including participants’ age, gender, sexual orientation, religion and profession. The second section comprised the Transgender Knowledge, Attitudes, and Beliefs (T-KAB) scale. The last section collected information on professional experience, including years in practice, any previously received training on providing services to transgender patients (without specification of training type, duration or content), and previous professional contact with transgender patients.

The T-KAB is a 22-item scale designed and validated to evaluate transgender-related knowledge, attitudes and beliefs across three domains: (1) acceptance and understanding of the gender spectrum (assessed by items 1, 2, 11, 12, 18, 19, 20 and 22), (2) social tolerance of transgender identity (assessed by items 3, 4, 5, 6, 7, 16 and 21), and (3) comfort and contact with transgender individuals (assessed by items 8, 9, 10, 13, 14, 15 and 17).^26^ The T-KAB scale was developed with expert input from transgender scholars, has demonstrated good convergent and discriminant validity, and has thus far been validated in the United States of America, Mexico and Portugal.^26,27,28^ While the T-KAB has not been formally validated in South Africa, it has demonstrated good psychometric properties across different cultural contexts and was administered in English, which is widely used in South African healthcare settings. Items are assessed via a 1–4 Likert scale.^26^ Higher total scores indicate greater knowledge and more accepting attitudes and beliefs towards TGD individuals. Permission to use the scale was obtained from the authors prior to commencement of this study.

### Statistical analysis

Statistical analyses were conducted using R software (version 4.0.0; www.R-project.org). Categorical data were reported as counts and percentages, and continuous data were reported as mean and standard deviation (s.d.). The fixed variables were all categorical, and comparisons of the components within each variable were analysed with Pearson’s chi-squared goodness-of-fit tests to assess whether the components differed from chance (i.e., a null model). Comparisons of the T-KAB scores for socio-demographic variables and for professional training and experience were analysed using Mann–Whitney *U* tests (*k* = 2 levels) or Kruskal–Wallis tests (*k* > 2 levels). Pairwise post hoc tests (with Bonferroni adjustments) were conducted following significant Kruskal–Wallis tests. Model significance was set at 0.05 and tests were two-tailed.

### Ethical considerations

Ethical approval for the study was obtained from the Wits Human Research Ethics Committee (reference number M240506-C-0001), and permission to conduct the study was obtained from the research committees of both Tara Hospital and Sterkfontein Hospital. Participation in the survey was voluntary, and all responses were anonymous, with no identifying information collected. There were no direct benefits for participants, and relevant information was provided on accessing psychological support if required for distress caused by completing the survey.

## Results

### Socio-demographic, professional and work experience characteristics of participants

A total of 150 mental healthcare workers participated in the study. All healthcare workers did not answer all socio-demographic or work experience questions in the study; in these few cases, the missing data were not considered in statistical analyses. There was a similar distribution between the two study sites: Sterkfontein Hospital (*n* = 79; 52.7%) and Tara Hospital (*n* = 68; 45.3%). Nurses (*n* = 92; 61.3%), followed by doctors (*n* = 41; 27.3%), comprised the majority of the study sample, with most respondents being 18–34 years old (*n* = 64; 42.7%). The socio-demographic and professional characteristics of the participants are shown in Table 1. Details on the professional work and training experience of the study participants are shown in Table 2.

**TABLE 1:** Socio-demographic and professional characteristics of mental health workers from Tara and Sterkfontein Hospitals who participated in the study.

Variable	*n*	%
**Profession**		
Nurse (professional and enrolled nurses)	92	61.3
Medical doctor (medical officer, registrar, specialist)	41	27.3
Psychologist (clinical psychologist and psychology interns)	6	4.0
Social worker	5	3.3
Occupational therapist (OT)	4	2.7
Did not specify	2	1.3
**Age (years)**		
18–34	64	42.7
35–44	47	31.3
45+	39	26.0
**Gender identity**		
Female	102	68.0
Male	46	30.7
Non-binary	1	0.7
Did not specify	1	0.7
**Sexual orientation**		
Heterosexual	117	78.0
LGBTQIA+	17	11.3
Did not specify	16	10.7
**Religion**		
Christian	109	72.7
Not religious	19	12.7
Other	21	14.0
Did not specify	1	0.7

LGBTQIA+, Lesbian, Gay, Bisexual, Transgender, Queer, Intersexual, Asexual and Others.

**TABLE 2:** Professional work and training experience of mental health workers from Tara and Sterkfontein Hospitals who participated in the study.

Variable	*n*	%
**Number of years of professional practice**		
1–4	39	26.0
5–9	49	32.7
10+	61	40.7
Did not specify	1	0.7
**Training for providing mental health services to transgender patients**		
Yes	30	20.0
No	119	79.3
Did not specify	1	0.7
**Previously provided mental health services to transgender patients**		
Yes	75	50.0
No	74	49.3
Did not specify	1	0.7

### Knowledge and attitudes of study participants towards transgender and gender-diverse individuals

#### Transgender knowledge, attitudes, and beliefs scores

On a scale of 1–4, with higher scores indicating more positive attitudes and beliefs towards TGD individuals, the mean T-KAB score among the study participants was 2.81 (s.d. ±0.71) for all 22 items of the T-KAB. In terms of the three domain subscales, the mean score was 2.57 (s.d. ±0.84) for the gender spectrum acceptance domain; 2.86 (s.d. ±0.81) for the social tolerance domain; and 2.87 (s.d. ±0.69) for the comfort and contact domain.

#### Factors associated with transgender knowledge, attitudes, and beliefs and domain scores

Analysis of socio-demographic characteristics revealed several significant associations with T-KAB scores in terms of age, sexual orientation and religious affiliation, but not gender identity. Professional category was also found to be significantly associated with all T-KAB measures (total score and domain scores), with psychologists showing the highest scores, followed by medical doctors, while nurses and social workers scored the lowest among the study participants. Professional work experience factors, including years of practice, previous training in transgender mental healthcare, and clinical exposure via having previously provided services to transgender patients, showed no significant associations with T-KAB scores. The relationship between T-KAB scores and socio-demographic characteristics of the study participants is shown in Table 3 and the relationship between T-KAB scores and professional, work and training experience is shown in Table 4.

**TABLE 3:** Relationship between the socio-demographic characteristics and transgender knowledge, attitudes, and beliefs scores (overall and domain) of mental health workers from Tara and Sterkfontein Hospitals who participated in the study.

Variables	T-KAB overall scores			Gender spectrum acceptance			Social tolerance			Comfort and contact		
	Mean	s.d.	Statistics	Mean	s.d.	Statistics	Mean	s.d.	Statistics	Mean	s.d.	Statistics
**Age (years)**	-	-	KW = 6.82, *df* = 2, ***p* = 0.033**	-	-	KW = 0.87, *df* = 2, *p* = 0.647	-	-	KW = 10.27, *df* = 2, ***p* = 0.006**	-	-	KW = 3.42, *df* = 2, *p* = 0.181
18–34	2.96	±0.68	-	2.63	±0.85	-	3.04	±0.71	-	2.97	±0.72	-
35–44	2.76	±0.74	-	2.54	±0.90	-	2.80	±0.83	-	2.86	±0.66	-
45+	2.62	±0.69	-	2.49	±0.76	-	2.58	±0.84	-	2.73	±0.67	-
**Gender identity**	-	-	W = 2579.5, *p* = 0.334	-	-	W = 2464.5, *p* = 0.624	-	-	W = 2617.5, *p* = 0.260	-	-	W = 2619.5, *p* = 0.257
Female	2.86	±0.69	-	2.59	±0.87	-	2.92	±0.77	-	2.93	±0.67	-
Male	2.71	±0.76	-	2.52	±0.80	-	2.73	±0.89	-	2.78	±0.72	-
**Sexual orientation**	-	-	KW = 20.85, *df* = 2, ***p* < 0.001**	-	-	KW = 11.44, *df* = 2, ***p* = 0.03**	-	-	KW = 19.63, *df* = 2, ***p* < 0.001**	-	-	KW = 18.62, *df* = 2, ***p* < 0.001**
Heterosexual	2.78	±0.70	-	2.51	±0.84	-	2.82	±0.82	-	2.87	±0.68	-
LGBTQIA+	3.42	±0.51	-	3.20	±0.68	-	3.52	±0.42	-	3.39	±0.56	-
Did not specify	2.37	±0.59	-	2.35	±0.72	-	2.44	±0.65	-	2.38	±0.56	-
**Religion**	-	-	KW = 9.75, *df* = 2, ***p* = 0.007**	-	-	KW = 8.52, *df* = 2, ***p* = 0.014**	-	-	KW = 8.37, *df* = 2, ***p* = 0.015**	-	-	KW = 10.50, *df* = 2, ***p* = 0.005**
Christian	2.71	±0.65	-	2.47	±0.76	-	2.77	±0.76	-	2.78	±0.62	-
Not religious	3.21	±0.81	-	3.10	±0.92	-	3.20	±0.97	-	3.29	±0.69	-
Other	2.89	±0.84	-	2.56	±1.01	-	2.99	±0.85	-	2.93	±0.89	-

Note: Significant outcomes are shown in bold.

Statistics: KW, Kruskal–Wallis tests; W, Mann–Whitney *U*-tests; T-KAB, transgender knowledge, attitudes, and beliefs; s.d., standard deviation; *df*, degrees of freedom; LGBTQIA+, Lesbian, Gay, Bisexual, Transgender, Queer, Intersexual, Asexual and Others.

**TABLE 4:** Relationship between professional work and training experience and transgender knowledge, attitudes, and beliefs scores (overall and domain) of mental health workers from Tara and Sterkfontein Hospitals who participated in the study.

Variables	T-KAB overall scores			Gender spectrum acceptance			Social tolerance			Comfort and contact		
	Mean	s.d.	Statistics	Mean	s.d.	Statistics	Mean	s.d.	Statistics	Mean	s.d.	Statistics
**Profession**	-	-	KW = 28.81, *df* = 4, ***p* < 0.001**	-	-	KW = 18.18, *df* = 4, ***p* = 0.001**	-	-	KW = 30.94, *df* = 4, ***p* < 0.001**	-	-	KW = 16.98, *df* = 4, ***p* = 0.002**
Psychologist	3.30	±0.59	-	2.94	±0.67	-	3.52	±0.69	-	3.31	±0.50	-
Medical doctor	3.23	±0.72	-	3.03	±0.92	-	3.31	±0.70	-	3.19	±0.78	-
Nurse	2.61	±0.61	-	2.36	±0.73	-	2.65	±0.74	-	2.73	±0.58	-
OT	2.85	±0.84	-	2.38	±1.10	-	3.07	±0.71	-	2.79	±0.97	-
Social worker	2.62	±0.93	-	2.73	±0.89	-	2.51	±1.03	-	2.69	±1.00	-
**Years of practice**	-	-	KW = 1.20, *df* = 2, *p* = 0.549	-	-	KW = 0.86, *df* = 2, *p* = 0.650	-	-	KW = 2.50, *df* = 2, *p* = 0.287	-	-	KW = 1.29, *df* = 2, *p* = 0.525
1–4	2.87	±0.59	-	2.64	±0.74	-	2.95	±0.71	-	2.95	±0.59	-
5–9	2.83	±0.84	-	2.48	±0.98	-	2.91	±0.92	-	2.91	±0.81	-
10+	2.75	±0.69	-	2.59	±0.79	-	2.77	±0.78	-	2.80	±0.66	-
**TGD training**	-	-	W = 1476, *p* = 0.144	-	-	W = 1464.5, *p* = 0.13	-	-	W = 1540, *p* = 0.246	-	-	W = 1392.5, *p* = 0.063
Yes	2.96	±0.70	-	2.74	±0.79	-	3.02	±0.79	-	3.06	±0.71	-
No	2.77	±0.72	-	2.52	±0.85	-	2.82	±0.83	-	2.83	±0.68	-
**Provided TGD services**	-	-	W = 2526, *p* = 0.347	-	-	W = 2553.5, *p* = 0.332	-	-	W = 2521.5, *p* = 0.337	-	-	W = 2422, *p* = 0.180
Yes	2.85	±0.72	-	2.57	±0.85	-	2.91	±0.81	-	2.94	±0.69	-
No	2.76	±0.71	-	2.56	±0.84	-	2.81	±0.81	-	2.81	±0.69	-

Note: Significant outcomes are shown in bold.

TGD, transgender and gender-diverse; T-KAB, transgender knowledge, attitudes, and beliefs; Statistics: KW, Kruskal–Wallis tests; W, Mann–Whitney *U* test; OT, occupational therapist; s.d., standard deviation.

## Discussion

This study found moderately favourable attitudes towards TGD individuals among mental health workers at two South African psychiatric hospitals. This finding is encouraging and noteworthy, representing one of very few studies examining the attitudes of mental health professionals towards TGD individuals in the Global South. Our mean T-KAB score of 2.81 is equivalent, or only slightly lower than those reported by several other studies using the T-KAB scale in groups of healthcare trainees and professionals in the United States that have reported mean total scores in the range of 2.89 to 3.61.^29,30,31^

Professional category emerged as a significant factor, with clinical psychologists and medical doctors demonstrating higher scores than nurses and social workers on the T-KAB scale. This finding has particular significance given that nurses constitute the majority of the mental health workforce and play a crucial role in both direct patient care and shaping institutional culture. Recent systematic review evidence highlights nurses’ critical role in shaping unit dynamics and patient experiences in inpatient psychiatric settings because of their constant presence and influence over the therapeutic environment.^13^ The attitudes and practices of nursing staff are therefore instrumental in determining patient experiences and establishing the overall environment of mental healthcare facilities.

Lower scores in the gender spectrum acceptance domain compared to social tolerance and comfort domains may reflect challenges with understanding gender diversity beyond traditional binary constructs. Notably, this domain relates more to underlying knowledge of gender constructs rather than attitudes towards individuals, suggesting potential benefit from educational interventions that specifically address these concepts.

The associations found between attitudes and personal characteristics such as age, sexual orientation, and religious affiliation align with previous research, which has also shown associations between younger ages, LGBTQIA+ identity and non-religiosity with more positive attitudes.^15,17,18,30^ The lack of association between attitudes and professional factors such as years of experience, previous training, or clinical exposure to transgender patients may reflect the mixed evidence in the literature on these relationships.^15,16^ Although there has been some evidence from systematic reviews that greater curricular training time may positively influence both knowledge and attitudes,^15^ our study found no significant associations between training and attitudes towards TGD patients. This discrepancy may reflect methodological differences in how these factors were measured or could suggest that other variables may be more influential in our specific setting. This finding should be interpreted in the light of recent systematic review evidence noting the lack of data on long-term impacts of educational interventions.^25^ In addition, we did not collect detailed information about the type and content of previous training our participants received, which the literature suggests can vary substantially. Further research should examine which training approaches are most effective for producing sustained improvements in attitudes and knowledge.

### Strengths and limitations

This study has several strengths, including the use of a standardised instrument to assess attitudes, and the inclusion of multiple professional categories across two psychiatric hospitals. To our knowledge, this represents one of the first assessments of mental health worker attitudes towards TGD individuals in an African psychiatric setting.

Several limitations should be considered when interpreting these findings. The study measured only explicit, self-reported attitudes, which may be influenced by social desirability bias and may not capture implicit biases that could affect patient care. The small sample size, cross-sectional nature of the study, and convenience sampling approach limit causal inference and generalisability. In particular, the very small numbers of allied health professionals (including psychologists, OTs and social workers) limited meaningful conclusions about these professional groups. In addition, the study may be affected by non-response bias, as those with more negative attitudes towards transgender individuals might have been less likely to participate in the survey. The T-KAB scale has not been formally validated in a South African context, which may affect the interpretation of scores, although it has been validated in multiple countries and contexts and is administered in English, which is the primary language of professional practice in South African psychiatric hospitals. Furthermore, we did not collect detailed information about the type and content of previous TGD training our participants received, which the literature suggests can vary substantially. Further research should examine which training approaches are most effective for producing sustained improvements in attitudes and knowledge. Finally, while attitude surveys provide valuable insight into individual-level factors, they cannot capture broader structural and systemic barriers to gender-affirming care. Recent evidence suggests that improving care for TGD individuals requires addressing multiple levels of stigma, including structural barriers such as institutional policies.^13^ The focus on individual attitudes, while important, should not detract from examining and addressing institutional and systemic factors that may impact access to inclusive mental healthcare.

### Recommendations

Further research is needed to understand effective approaches to improving knowledge and attitudes among mental health workers, particularly among nursing populations, and with a potential focus on educational interventions that address fundamental understanding of gender diversity. Future studies should also examine how individual attitudes interact with structural barriers in determining patient experiences of mental healthcare.

## Conclusion

This study reveals moderately positive attitudes towards TGD individuals among South African mental health workers, with important variations across professional categories that have implications for training. Future efforts should focus on improving understanding of gender diversity concepts, particularly among nursing staff who constitute the majority of the mental health workforce and play a crucial role in shaping patients’ experiences in psychiatric settings.
